# Correction: Predicting unsafe behaviour from the objective assessment of fatigue manifestation among scaffolders: Evidence from a Quasi-experimental simulation study

**DOI:** 10.1371/journal.pone.0342174

**Published:** 2026-01-30

**Authors:** Pei Pei Heng, Hanizah Mohd Yusoff, Mohamad Ridza Hj Illias, Jun Fai Yap, Muhammad Fadhli Mohd Yusoff, Norizzati binti Amsah, Rozita Hod

The fourth author’s name is spelled incorrectly. The correct name is: Jun Fai Yap. The correct citation is: Heng PP, Yusoff HM, Hj Illias MR, Yap JF, Yusoff MFM, Amsah Nb, et al. (2026) Predicting unsafe behaviour from the objective assessment of fatigue manifestation among scaffolders: Evidence from a Quasi-experimental simulation study. PLoS One 21(1): e0339055. https://doi.org/10.1371/journal.pone.0339055
