# Supplementary material for: Predicting unsafe behaviour from the objective assessment of fatigue manifestation among scaffolders: Evidence from a Quasi-experimental simulation study
Source: PLoS One. 2026 Jan 2;21(1):e0339055. doi: 10.1371/journal.pone.0339055 (PMC12758696; doi:10.1371/journal.pone.0339055)
Supplement: S1 Table — (DOCX) [file pone.0339055.s001.docx]

**SUPPLEMENTARY**

**S1 Table. Validated Checklist for Individual Evaluation of Unsafe Behavior during the 2-Lifts Basic Static Scaffold Tower Erection Protocol**

| **No** | **Task** | **Unsafe behavior** | **Complied (/)**  **Not-complied (X)** |
| --- | --- | --- | --- |
|  | 1. **INSTRUCTION (After briefing and before materials collection)** |  |  |
| 1 | Wear full set of Personal Protective Equipment | Wear single lanyard instead of double lanyard |  |
| 2 | Look for water level ruler, torque and measuring tape, marker pen and carry with them | Do not bring along water level ruler, torque, measuring tape and marker pen |  |
|  | 1. **PREPARATION (At storage area while selecting materials)** |  |  |
| 3 | Inspect materials for defect | Selection of incompatible materials (crooked, rusty, or broken) |  |
| 4 | Two persons to lift the standard (the vertical rod) from storage area to site | One person lifts the standard alone |  |
|  | 1. **FOUNDATION (At the site)** |  |  |
| 5 | Measure distance between 4 sole boards using measuring tape; and place ledger and transom (the horizontal tubes) on the floor between all sole boards | Do not measure the distance between sole boards. |  |
| 6 | When supporting the standard, both hands are used at all time | Support with one hand only |  |
| 7 | When supporting the standard, if tired, to rest the standard on shoulder | Directly put the standard on the floor |  |
| 8 | If fail to support the standard, ask for help from co-workers | Unable to maintain position causing the fall or collapse of standard |  |
| 9 | When fixing the coupler, the tighten part of coupler must face upward.  * Coupler is fixed to hold the scaffold rods together | Coupler fixed at wrong facing |  |
| 10 | Tighten coupler adequately and counter check the tightening with 54Newton meter torque | Not using torque when checking the tightening of coupler, or only check based on the “click” sound |  |
| 11 | After 4 standards being fixed, counter check the measurement of ledgers and transom (the horizontal rods), and the oblique distance. | Skip the measurement |  |
| 12 | Check the horizontality of the horizontal rod using water level ruler | Checking base on eye level |  |
| 13 | Check the verticality of the vertical rod with water level ruler | Checking base on eye level |  |
| 14 | Check all four standards to be placed in the middle of sole board | Skip the counter check |  |
|  | 1. **CONSTRUCTION OF 1^ST^ LIFT** |  |  |
| 15 | After measuring the height and do marking at the standard with marker pen, lift up the 4 horizontal rods that have been fixed to coupler at foundation, until they reach the marking points. | When lifting up the horizontal rod, there is slip of rod or the holding is not stable |  |
| 16 | When fixing the coupler, the tighten part of coupler must face upward.  (* Coupler is fixed to hold the scaffold rods together) | Coupler fixed at wrong facing |  |
| 17 | Tighten coupler and counter check the tightening with 54Newton meter torque | Not using torque when checking the tightening of coupler, or only check based on the “click” sound |  |
| 18 | Check the horizontality of the horizontal rod using water level ruler | Checking base on eye level |  |
| 19 | Check the verticality of the vertical rod with two water level rulers | Checking base on eye level, or only use one water level ruler |  |
| 20 | When fixing the four bracing (oblique rod), the tighten part of coupler must face externally | Coupler fixed at wrong facing |  |
|  | **(E)CONSTRUCTION OF WORKING PLATFORM** |  |  |
| 21 | Lift up and place 4 boards as the working platform of 2nd lift, 2 boards at each end, and stabilize every two boards using mushroom coupler. | Skip the usage of mushroom coupler |  |
| 22 | Take the 3-meter ladder to access to the working platform | Directly climb up to the lift without using ladder or using the overlength ladder |  |
| 23 | Secure ladder with 4 wires, 2 at the top and 2 at the bottom | Do not secure ladder  Secure ladder with less than 4 wires |  |
| 24 | Once reach up platform, maintain in squatting position until the guardrail is completely constructed | Standing on platform |  |
| 25 | Secure double lanyard temporarily at the Advanced Guardrail System (AGS) | Do not secured lanyard, or only secure single lanyard |  |
| 26 | Hook double lanyard at the horizontal rod | Hook double lanyard at the vertical rod |  |
|  | **(F)INSTALL GUARDRAILS** |  |  |
| 27 | Receive materials from below | Slip and fall of material or loss of balance |  |
| 28 | When fixing the coupler, the tighten part of coupler must face upward.  (* Coupler is fixed to hold the scaffold rods together) | Coupler fixed at wrong facing |  |
| 29 | Tighten coupler and counter check the tightening with 54Newton meter torque | Not using torque when checking the tightening of coupler, or only check based on the “click” sound |  |
|  | **(G)CONSTRUCTION OF 2^ND^ LIFT** |  |  |
| 30 | Measure the height from then base of second lift to the top of second lift with measuring tape and do the marking | Skip the measurement or skip the marking |  |
| 31 | Receive rods and couplers from below | Slip and fall of material or loss of balance |  |
| 32 | When fixing the coupler, the tighten part of coupler must face upward.  (* Coupler is fixed to hold the scaffold rods together) | Coupler fixed at wrong facing |  |
| 33 | Tighten coupler and counter check the tightening with 54Newton meter torque | Not using torque when checking the tightening of coupler, or only check based on the “click” sound |  |
| 34 | When the top of 2^nd^ lift is readily constructed, change the hook of double lanyard from lower horizontal rod to newly constructed upper horizontal rod | Do not change the hook of double lanyard from lower horizontal rod to newly constructed upper horizontal rod |  |
| 35 | At all time, the feet must be maintained within the safe zone which is surrounded by guardrails | Feet placed beyond the safe zone |  |
| 36 | Receive 8 boards from below | Slip or fall of material or loss of balance |  |
| 37 | Lift up and place 8 boards as the working platform of 2nd lift, and tie every two boards together with wire, making up a total of 4 ties on the left and 4 ties on the right | Skip the tie, or make up less than 4 ties on the left and right |  |
|  | **AFTER TASK COMPLETION** |  |  |
| 38 | Climb down the scaffold platform using ladder | Do not use ladder |  |
